# Calculus at the duct orifice of the submandibular gland in a patient with an edentulous jaw: A case report and literature review

**DOI:** 10.1097/MD.0000000000043020

**Published:** 2025-07-04

**Authors:** Taiqi Cheng, Zhiyu Ma, Xing Yan

**Affiliations:** a Department of Stomatology, Beijing Friendship Hospital, Capital Medical University, Beijing 100050, China.

**Keywords:** disease prevention, edentulous jaw, sialolithiasis, submandibular salivary gland

## Abstract

**Rationale:**

The intricate and uncontrollable etiology of sialolithiasis compels clinicians to focus solely on disease management, often overlooking the underlying causes of sialolithiasis and neglecting patient education regarding preventive measures. The use of the multidisciplinary Salivary Gland Association questionnaire enables the identification of benign salivary gland lesions and evaluation of patients’ quality of life.

**Patient concerns:**

Wanting to remove foreign body from the bottom of the right lower side of the mouth.

**Diagnoses:**

Ductal calculus of the right submandibular gland.

**Interventions:**

We present a case of calculi in the right submandibular duct orifice of a 66-year-old male patient with an edentulous jaw. The patient reported discovering foreign bodies on the floor of the mouth persisting for 1 week without experiencing any discomfort and requested treatment for these foreign bodies. Subsequently, the patient underwent a transoral sialolith extraction.

**Outcomes:**

The patient experienced no postoperative discomfort, and the submandibular gland texture and salivary secretion function were normal after 8 weeks of follow-up. The patient’s Multidisciplinary Salivary Gland Society (MSGS)/Q10 score decreased significantly 6 months after surgery.

**Lessons:**

Sialolithiasis may remain asymptomatic throughout its course, and early intervention and treatment can effectively prevent severe complications. The MSGS/Q10 questionnaire may be a valuable tool.

## 1. Introduction

Sialolithiasis is a benign condition characterized by the formation of stones in the ducts or parenchyma of salivary glands. It presents as swelling or pain in the submandibular area after meals. If left untreated, it can progress to salivary gland inflammation, fibrosis, and other pathological manifestations, ultimately resulting in impaired salivary gland function.^[1]^ This report describes a case of asymptomatic right submandibular duct calculus in an edentulous patient who underwent effective treatment. This study aimed to describe the etiology and management of sialolithiasis and propose early diagnosis and treatment for high-risk individuals through self-questionnaires to prevent serious complications. This case report conforms to the SCARE 2023 criteria.^[2]^

## 2. Case presentation

A 66-year-old man presented to a medical institution with a complaint of sensation of a foreign body on the floor of the mouth persisting for 1 week. The patient reported the texture to be firm upon self-examination, without any associated discomfort such as pain or numbness. There was no history of recurrent pain or swelling in the submandibular gland region. The patient sought treatment. The patient was edentulous and had been using complete dentures for the past decade. The self-reported prosthesis caused no discomfort and demonstrated satisfactory masticatory function. After being informed of the potential risks, the patient refused to undergo routine postoperative pathological examinations and subsequently provided informed consent.

**Figure 1.**
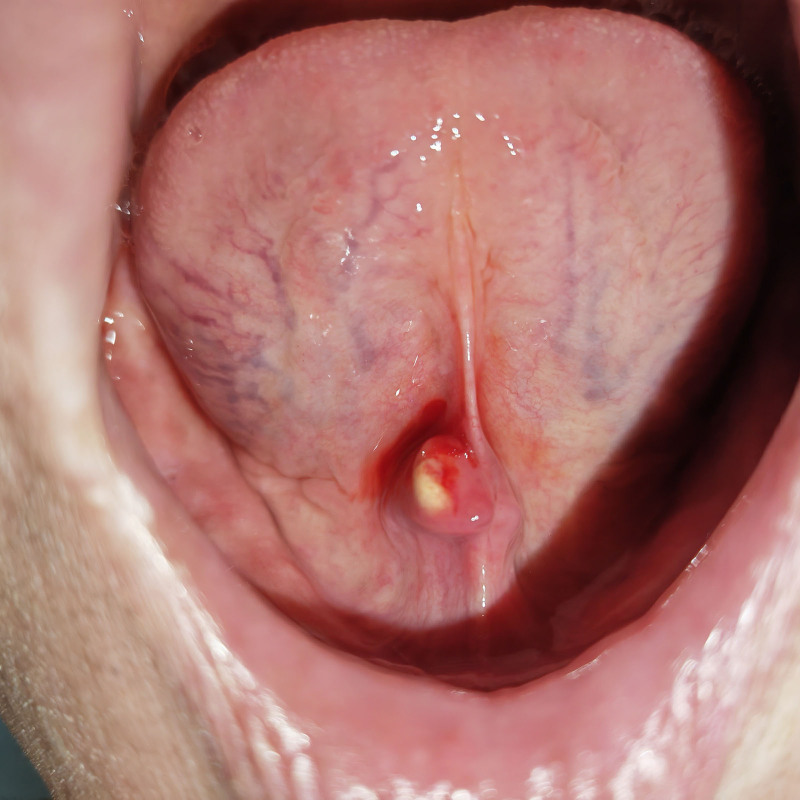
The image reveals swelling near the mandible, with a thin mucosal surface and reflective white content.

Local examination revealed a circular mass measuring approximately 10 mm in diameter containing white solid and uniform contents located 1 mm posterior to the catheter opening of the right submandibular gland (Fig. 1). Central mucosal perforation was observed, with a small amount of bloody fluid visible upon contact with the tongue. The bilateral submandibular and sublingual glands exhibited a soft texture without tenderness. Extrusion of the submandibular gland resulted in clear fluid drainage from the left catheter opening but not from the right side. Maxillofacial computed tomography (CT) or ultrasound examinations were not performed due to economic considerations.

**Figure 2.**
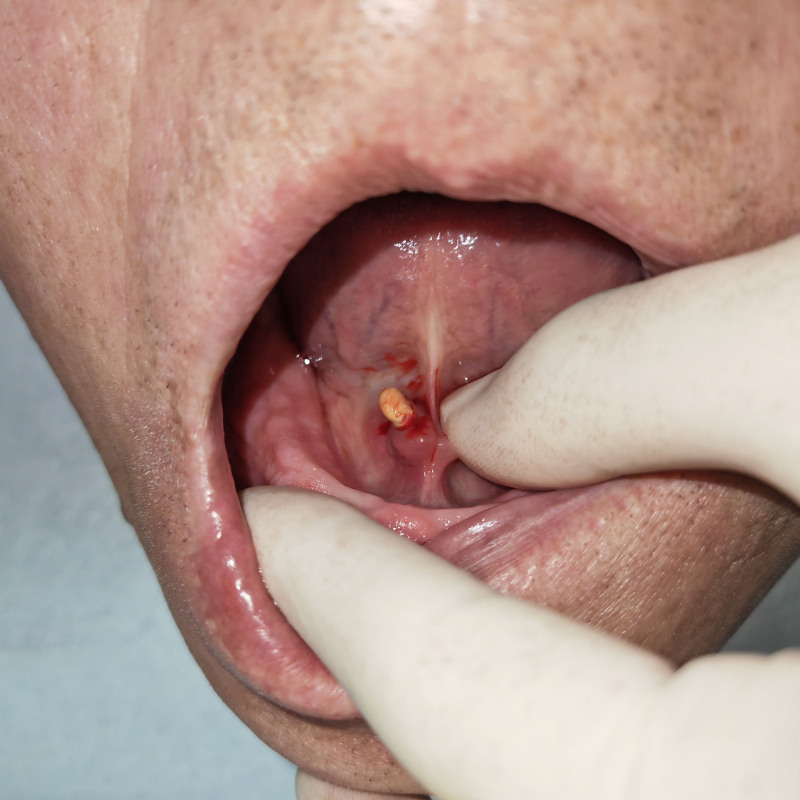
The perforation is damaged, resulting in the discharge of a sialic acid stone and the expulsion of a significant amount of clear liquid from the perforation at the moment of discharge.

**Figure 3.**
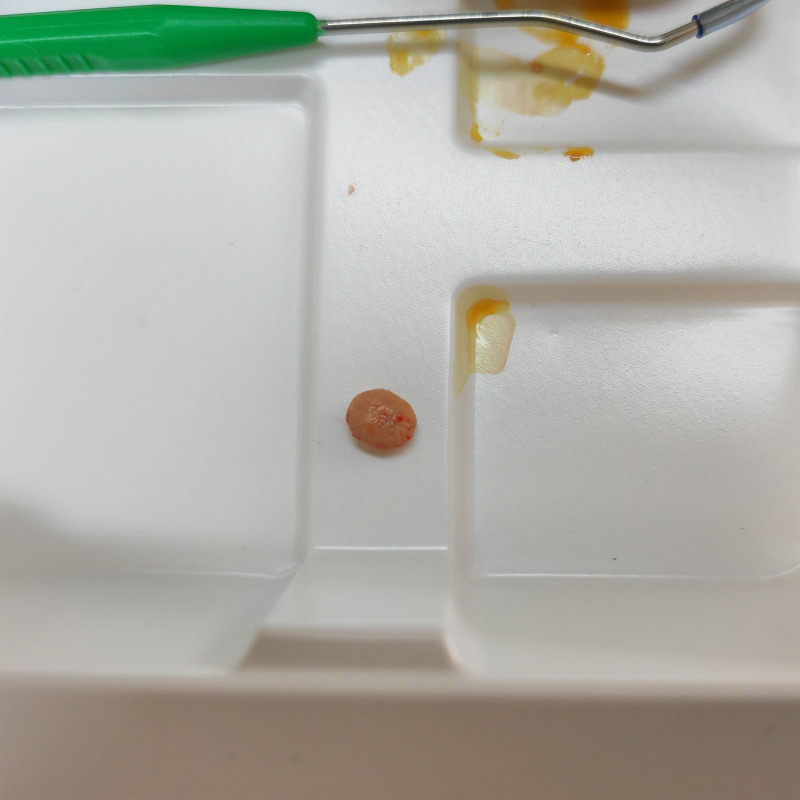
The dimensions of the sialoliths in the submandibular gland are 9 mm × 7 mm × 5 mm.

**Figure 4.**
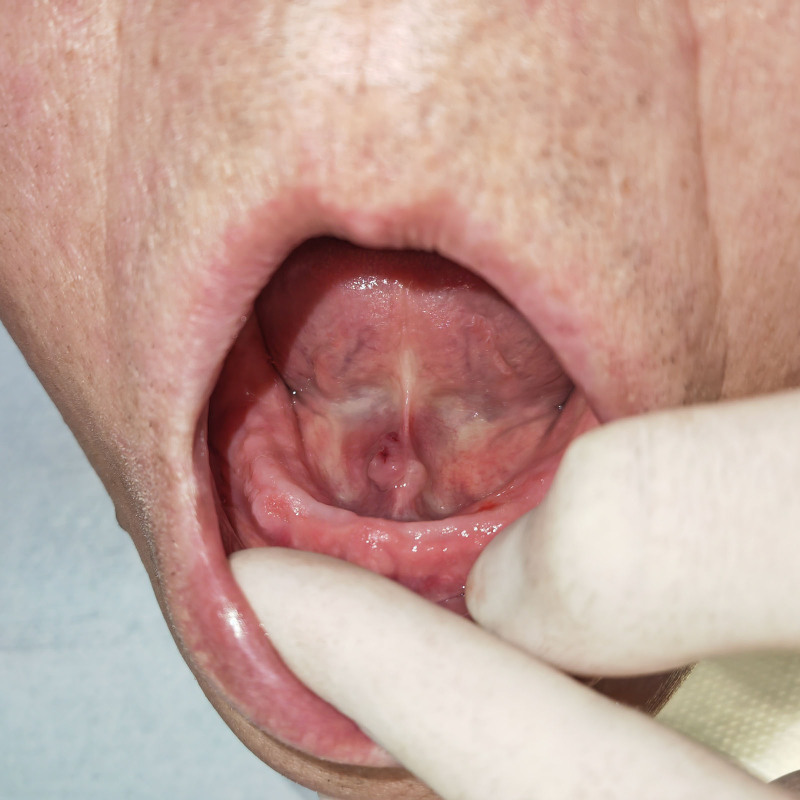
After extracting the sialolith from the right submandibular gland, a perforation located 1 mm away from the catheter opening was observed.

**Figure 5.**
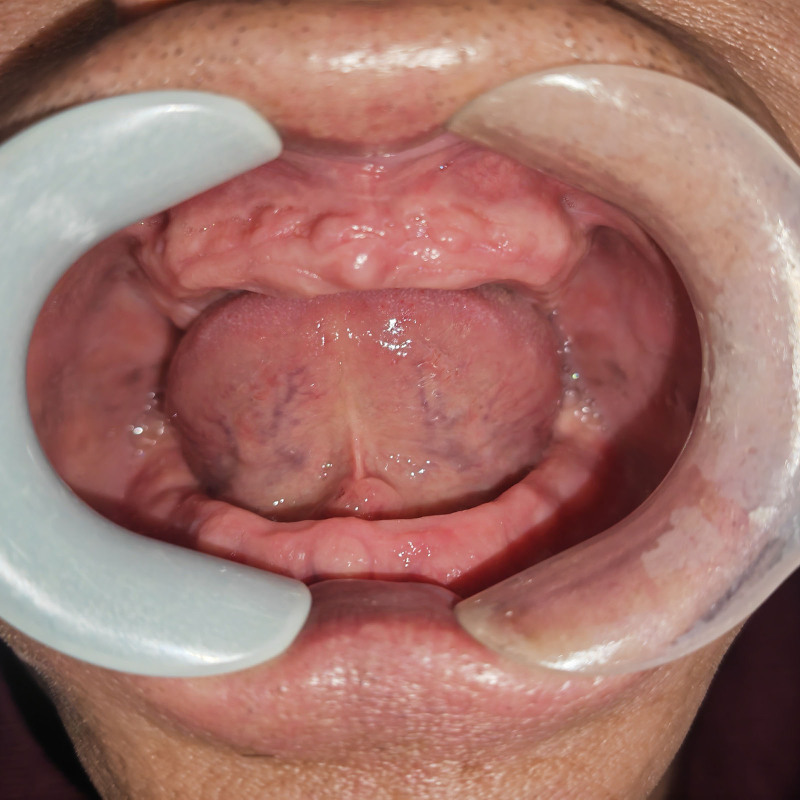
After 8 wk of follow-up, edema of the sublingual area reduced, the perforation underwent epithelialization, and secretory function normalized.

After administering local anesthesia, we dilated the catheter orifice of the right submandibular gland to perform an enlarged catheter sialoliths extraction procedure. However, during the process of enlarging the catheter orifice, the mucosal perforation on the tumor surface got damaged (Fig. 2). Therefore, we switched to transoral sialolith extraction and successfully removed the ellipsoid contents measuring 9 mm × 7 mm × 5 mm (Fig. 3). Considering that the patient had a narrow catheter opening and that the perforation was <1 mm away from it (Fig. 4), we decided not to perform suturing to avoid stenosis caused by scar contracture after surgery. Continuous telephone follow-ups were conducted once a week after surgery to monitor any signs of swelling, pain, dry mouth, or other discomfort. After 8 weeks, the patient returned to the medical institution for a clinical visit, where it was observed that the mucosal perforation at the bottom of the mouth had epithelialized and healed completely (Fig. 5). Additionally, a significant amount of fluid flowed out from the squeezed glands, indicating normal texture and salivary secretion from the submandibular glands. No abnormalities were observed 6 months postoperation.

## 3. Discussion

More than 80% of sialolithiasis cases occur in the submandibular gland, predominantly affecting individuals aged 40 to 60, with a higher prevalence rate observed in men.^[1,3]^ This often results in the obstruction of the submandibular gland catheter and chronic inflammation of the salivary glands accompanied by symptoms such as swelling, pain, impaired salivary secretion, or secondary infections.^[3]^

Currently, the etiology of sialolithogenesis remains unclear. The prevailing belief among scholars is that when bacterial infection occurs in the submandibular gland duct or gland, the bacteria will encapsulate salivary mucins and shed epithelial cells in the saliva to form a mineral salt deposition core because of the high content of inorganic salts such as calcium and phosphorus. Simultaneously, the anatomical structure of the submandibular gland duct, which allows for slow salivary secretion from the bottom up, leads to prolonged existence of the deposition core within the glandular substance or duct.^[4]^ Consequently, numerous layers of inorganic salts gradually deposit around this core, resulting in sialoliths of varying sizes. Some scholars argue that considering the location of the opening of the submandibular gland duct and its large cavity structure, ingestion and mastication can introduce food residues and other external organic nuclei into the submandibular gland duct, serving as a core for sialolith formation.^[5,6]^ Consequently, localized sialoliths within the duct are more prevalent than those within the glandular parenchyma.^[7]^ Furthermore, smoking, salivary stasis, salivary gland trauma, infection, etc, serve as precursors to sialolithiasis.^[4,8]^

Currently, multiple diagnostic methods are available for sialolithiasis, with a clinical examination of the base of the mouth being the initial step. Approximately 75% of submandibular gland sialoliths are unilateral, whereas only 3% are bilateral. Most patients present with complaints of a mass in the submandibular area or experience swelling and pain in that region after eating. Most stones located at the front part of the catheter can be easily detected by applying downward pressure to the submandibular gland towards the bottom of the mouth in the submaxillary region using 1 hand and employing sliding palpation from the front catheter with the index finger of the other hand. However, accurately diagnosing stones situated at the back and within the glandular tissue remains challenging. Therefore, auxiliary examinations such as radiography, ultrasonography, CT, cone-beam CT, magnetic resonance imaging, and sialography are also employed to evaluate the presence and location of sialoliths.^[3,9,10]^ In the clinical diagnosis of sialolithiasis, the patient’s condition and clinical examination findings typically serve as the primary diagnostic indicators. Additionally, personalized auxiliary examination protocols tailored to individual patients can enhance the accuracy of sialolithiasis diagnosis.

Currently, there are various clinical treatment modalities for sialolithiasis, including conservative management, expanded catheter-based sialolith extraction, endoscopic sialolith extraction, transoral sialolith removal, extracorporeal shock wave lithotripsy, laser lithotripsy, transoral submandibular gland excision, and extracoronal submandibular gland excision.^[1,3,10,11]^ The primary determinants for selecting from the different surgical approaches include the location of the calculi (within the duct or glandular parenchyma), size and shape of the stones, mobility of the stones within the salivary system, extent of inflammatory infiltration in the salivary glands (degree of inflammation), local symptoms (e.g., swelling, pain, infection, or fistula formation), and the overall general condition (tolerance to anesthesia and surgery). Below is a list outlining common lithotomy procedures that can be considered based on the site and size of the calculi (Table 1).^[1,3,10,12,13]^ (Supplement: for stones larger than 8 mm in diameter, ultrasonic lithotripsy often fails to achieve complete stone clearance and may leave residual fragments within the catheter which could serve as potential foci for recurrent sialolithiasis. Stones larger than 5 mm in diameter, particularly irregularly shaped ones, are prone to endoscopic complications, such as catheter perforation.)

**Table 1:** Enhanced comparative analysis of the standard treatment methods for sialoliths in various locations and sizes.

Sialolith position	Sialolith size	Removal method	Reference
Distal region of duct	<5 mm	1	Takahara et al^[1]^ Badash et al^[3]^ Abraham et al^[10]^ Quiz and Gillespie^[12]^ Ungari et al^[13]^
	5–8 mm	1	
	>8 mm	1 or 3	
Proximal region of duct	<5 mm	2 or 3 or 4	
	5–8 mm	3 or 4	
	>8 mm	3	
Gland parenchyma	<5 mm	2 or 3 or 4 or 5	
	5–8 mm	3 or 4 or 5	
	>8 mm	3 or 5	

The selection of common lithotomy techniques for the extraction of different sialoliths depends on their location and size. The methods listed in the table include: 1, expanded catheter-assisted sialolith extraction; 2, endoscopic sialolith extraction; 3, transoral sialolith extraction; 4, extracorporeal shock wave lithotripsy; and 5, salivary gland removal.

The presence of large calculi in the ductal orifice of the submandibular gland in an edentulous mandible was reported for the first time in this case. Although large or giant sialoliths are frequently observed in clinical practice, multifactorial and complex etiological conditions often lead clinicians to focus solely on disease treatment, disregarding sialolith formation and prevention education. Dry mouth is more prevalent among middle-aged and older individuals, and tooth loss further exacerbates saliva secretion issues, thereby increasing the risk of dry mouth in these populations.^[14]^ Complete denture restoration not only restores chewing ability but also significantly enhances saliva secretion and alleviates dry mouth symptoms. For prosthodontists, adequate salivary secretion improves complete denture retention and functionality.^[15]^ Similarly, oral and maxillofacial surgeons may benefit from sufficient salivation because it potentially reduces the risk of developing sialolithiasis to some extent. Although treatment options exist for submandibular gland stones in patients with edentulous jaws, as in the present case study, accurately assessing the high-risk factors and severity associated with submandibular gland stone formation remains challenging for middle-aged and older individuals without teeth, particularly those with edentulous jaws. Based on a growth rate of 1 mm per year (the diameter of the patient’s sialoliths was approximately 9 mm) combined with a history of tooth loss spanning 10 years, it can be inferred that the sialoliths formed shortly after tooth loss in this patient. Although there is no direct evidence linking toothless jaws to the development of sialolithiasis, this case provides valuable insights for clinicians.

In addition, numerous researchers have conducted questionnaire surveys to assess the efficacy of treatments for xerostomia or submandibular adenitis, and the research findings consistently indicate that questionnaires provide a more accurate evaluation of patients’ disease status and treatment outcomes.^[16–18]^ Recently, researchers introduced the concept of patient-reported outcomes (PROs), which refers to direct reports from patients regarding their specific health conditions through professional interviews covering 15 aspects of life before and after chronic salivary gland treatments. Three validated subscales focusing on physical symptoms, psychosocial symptoms, and activity limitation were established. The SCARE 2023 guidelines also emphasize the significance of patient-centered care.^[2]^

The patient in this case did not show salivary gland inflammation throughout the disease course, and a significant volume of clear fluid was observed after sialolith removal. Considering the practical challenges encountered in clinical practice, limited doctor–patient communication fails to accurately reflect the impact of disease conditions on patients’ quality of life. Therefore, it is imperative to develop and implement patient-centered measures for self-perception. Similarly, incorporating dynamic questionnaire reports during disease diagnosis and treatment can facilitate risk factor assessment and the development of prevention and management strategies, making their application essential in clinical settings. Recently, the Multidisciplinary Salivary Gland Society (MSGS)/Q10 version of the questionnaire has been recommended for precisely evaluating benign salivary gland lesions (e.g., xerostomia and salivary gland inflammation) and patients’ quality of life.^[19]^ The MSGS/ Q10 questionnaire was administered to the patient both preoperatively and 6 months postoperatively. The results indicated a reduction in the patient scores from 34 to 13. Notably, there was a significant decrease in scores related to nocturnal water intake upon awakening, saliva taste, and mealtime swelling in the salivary gland region. Although the questionnaire is unable to particularly predict sialolithiasis occurrence, it consistently distinguishes patients from healthy individuals, and prospective multicenter studies utilizing this questionnaire are currently underway.

## 4. Conclusion

Sialolithiasis predominantly affects middle-aged and older individuals and may be asymptomatic during both the early and advanced stages. However, a lack of symptoms does not necessarily indicate the absence of sialoliths. Clinicians can employ the MSGS/Q10 questionnaire to perform personalized risk assessments for varying degrees of risk factors (e.g., age, tooth loss, autoimmune diseases, and medication history) in different patients. Timely identification, prompt diagnosis, and early intervention should be implemented in high-risk patients or those suspected of having sialolithiasis to prevent further complications including salivary gland inflammation, fibrosis, and even glandectomy.

## Acknowledgments

Thanks to Beijing Friendship Hospital Affiliated to Capital Medical University for their support to write this case report.

## Author contributions

**Conceptualization:** Taiqi Cheng, Xing Yan.

**Data curation:** Taiqi Cheng.

**Project administration:** Xing Yan.

**Supervision:** Xing Yan.

**Validation:** Taiqi Cheng, Zhiyu Ma.

**Writing – original draft:** Taiqi Cheng, Zhiyu Ma.

**Writing – review & editing:** Taiqi Cheng, Zhiyu Ma, Xing Yan.
